# A multi-agent system for distributed multi-project scheduling with two-stage decomposition

**DOI:** 10.1371/journal.pone.0205445

**Published:** 2018-10-09

**Authors:** Feifei Li, Zhe Xu

**Affiliations:** School of Economics and Management, Beihang University, Beijing, China; Shandong University of Science and Technology, CHINA

## Abstract

A two-stage decomposition approach based on a novel multi-agent system (MAS) is proposed for the distributed resource constrained multi-project scheduling problem (DRCMPSP). In stage one, from the point of view of each local project manager, a forward-backward hybrid genetic algorithm (FBHGA) is developed to generate an initial local schedule with the objective of minimizing individual project makespan. In stage two, from the global perspective of project management office, a sequential game-based negotiation mechanism is employed to eliminate global resource conflicts with the objective of minimizing total tardiness cost (*TTC*). The proposed approach is tested on 140 benchmark problem instances. According to the computational results, high-quality local project schedules can be obtained by FBHGA in stage one. Furthermore, it is observed that our method is capable of dealing with various complex multi-project instances under different degrees of resource conflicts in reasonable CPU running time. Compared to the existing decentralized methods for DRCMPSP, the proposed approach with sequential game-based negotiation mechanism shows the superiority in producing multi-project schedules with lower *TTC*, especially for large-size and strong conflicting instances.

## Introduction

The resource-constrained multi-project scheduling problem (RCMPSP), as a generalization of the traditional resource-constrained project scheduling problem (RCPSP) [1], is more pervasive in today’s enterprise project management. According to Turner [2], at least 90% of projects are executed in a multi-project environment. Indeed, more and more companies are likely to manage more than one project simultaneously for the purpose of lower management cost and shorter completion time. As mentioned by Payne [3], managing multiple projects at the same time could bring tremendous benefits for the business firms. Moreover, the current multi-project context is becoming more and more distributed with the speedy development of Internet technology and globalization [4], which means that the multiple projects could be located at different places and each project is managed by an autonomous decision maker (i.e., local project manager). In this circumstance, local project managers are responsible for scheduling all activities within their respective projects independently. Consequently, the distributed resource constrained multi-project scheduling problem (DRCMPSP) has been put forward formally [5].

A great deal of multi-project scheduling problems in service operations and manufacturing are managed and run in a distributed way. Examples can be commonly found in maintenance services [6,7] and supply chain management [8–10]. How to determine the right location for shared storage facilities and allocate the limited global resources to each project is vital for achieving the success of multi-project scheduling with resource transfers [11]. In the past, RCMPSP is mainly solved by centralized methods, which focus on allocating resources and scheduling activities for all projects by only one decision maker in an integrated way [12,13]. Specifically, multiple projects are combined into a single mega-project with two dummy activities which represent the start and end of all the projects respectively. Compared to the integrated means, DRCMPSP dedicates to making decisions in a distributed manner. To be more specific, all projects running parallel in different sites are performed by different decision makers i.e., the local project managers. Given the distributed nature, DRCMPSP involves the coordination of shared global resources among multiple projects. Owing to the assumption of asymmetric distribution of information in DRCMPSP, multi-agent systems (MAS) are used to make distributed decisions [14]. A MAS represents a decentralized system including a set of independent, autonomous and self-interested agents [15]. It is prevalent that companies adopt the dual level management structure for multi-project scheduling: the project management office (i.e., senior manager) who controls the resource allocation and coordination among the rival projects, and the project managers who are responsible for the specific affairs of each project such as scheduling [16]. Therefore, agents playing the roles of senior manager and project manager should be considered in MAS. Furthermore, the coordination mechanism for shared resources allocation is the key to solving DRCMPSP.

In this paper, we employ a two-stage decomposition approach based on a new MAS for DRCMPSP. It corresponds to the dual level managerial mechanism in actual enterprises. In stage one, each project manager is devoted to completing all tasks as soon as possible. In stage two, the senior manager acts as a coordinator agent and is mainly responsible for eliminating the resource conflicts among competing projects by a novel sequential game-based negotiation mechanism. Taking into account the delay penalty such as customer complaints and reputation loss, a senior manager usually attaches more importance to the total tardiness cost (*TTC*) rather than the most regular and popular measure of time performance (e.g. average project delay (*APD*)) in practical multi-project management scenarios [17]. Therefore, we develop the forward-backward hybrid genetic algorithm (FBHGA) to generate an initial local schedule with the goal of minimizing project makespan for each project manager in stage one, and design a sequential game-based negotiation mechanism to coordinate the global resources allocation for the senior manager in stage two where the best subgame perfect Nash equilibrium is determined with minimized *TTC* after several iterations. In order to test the proposed approach, we implement computational experiments on the instances from MPSPLIB problem library for DRCMPSP [18]. The results given by FBHGA in stage one are compared to the optimal solutions given by the branch-and-bound (B&B) algorithm embedded in RESCON [19]. To illustrate the strength of the two-stage decomposition approach with sequential game-based negotiation mechanism, we also compare our approach with two state-of-the-art decentralized methods with respect to the objective of minimizing *TTC*.

The remainder of this paper is structured as follows: an overview of the previous work on DRCMPSP is provided in Section 2, followed by a detailed description of the problem discussed in this paper (Section 3). In Section 4, we introduce the agent-based two-stage decomposition approach. Section 5 demonstrates an illustrative example. Section 6 explores the experiments and analyzes the computational results. Concluding remarks are drawn in the last Section 7.

## Literature review

Resource-constrained project scheduling problem (RCPSP), as one of the most complicated problems of operation research, has gained significant importance in the past few years. It is a classical and well-known standard problem in project management [20]. As a generalization of the job-shop scheduling problem, it is NP-hard in the strong sense [21]. Demeulemeester & Herroelen [22] proposed a depth-first branch-and-bound algorithm to solve the exact optimal solutions for smaller instances. A large number of heuristic and meta-heuristic procedures have been developed to obtain the approximate optimal solutions for large-scale problems. Kolisch [23] presented the priority rule-based approached for RCPSP and evaluated the performance of different priority rules. Lova & Tormos [12] analyzed the effect of serial and parallel schedule generation schemes with five priority rules for single project and multi-project instances, respectively. According to the computational study, the parallel scheduling generation scheme was superior for multi-project scheduling problem. Gonçalvesabc [24] and Mendes et al. [25] employed a genetic algorithm (GA) for RCPSP where the chromosome representation was based on the random keys. Moreover, the simulated annealing (SA) [26], tabu search (TS) [27] and ant colony optimization (ACO) [28] algorithms were also widely used for RCPSP. Hartman & Kolisch [29] summarized and categorized a large of heuristics in the RCPSP literature. They also evaluated the performance of several state-of-the-art algorithms and examined the impact of problem characteristics on it. The experimental investigation was updated a few years later [30].

While a large body of public literature addresses RCPSP, less importance has been attached to the multiple projects cases. It goes without saying that the great majority of papers on multi-project scheduling problem concentrate on allocating shared resources in an integrated manner where the multiple projects are controlled by only one manager [12,31,32]. In light of the distributed environment of multi-project management in the real world, studies on DRCMPSP have attracted increasing attention in the last decade. Traditional approaches to RCMPSP are no longer applicable to DRCMPSP owing to the informational decentralization. MAS is generally used to formulate DRCMPSP. Moreover, different coordination mechanisms for global resources allocation will exert a significant effect on the quality of solutions.

Some of the existing studies are committed to market-based mechanisms. Lee et al. [4] put forward a dynamic economy model, where a market-based control mechanism was developed to allocate limited shared resources for each required project to minimize *APD*. Confessore et al. [5] formally distinguished between local resources and global resources. An iterative combinatorial auction mechanism was proposed to address the global resource conflicts. Unfortunately, above-stated approaches were only suitable for small problems with the assumption that all global resources were single-unit. Araúzo et al. [33] presented a combinatorial auction mechanism to allocate the limited resources for project portfolio. Only global resources were considered. Adhau et al. [34] proposed a multi-unit combinatorial auction-based negotiation approach (DMAS/ASN). To minimize *APD*, three cost elements were considered in calculating the bidders’ utility. The authors further extended the above model with the assumption that the cost and time in transferring global resources could not be ignored [35]. Song et al. [36] resolved the DRCMPSP based on the multi-unit combinatorial auction method and the global resource conflicts were coordinated on project level instead of activity level. In addition to the above-mentioned market-based mechanisms to address DRCMPSP, negotiation based approaches have also gained tremendous popularity. Homberger [37] proposed a MAS-based restart evolution strategy to coordinate the allocation of global resources. Several scheduling agents with a mediator agent were contained in the MAS. On the basis of this work, the author presented a generic negotiation-based mechanism to solve all the problem instances in MPSPLIB problem library with the goal of minimizing *APD* [18]. Wauters et al. [38] introduced a learning-based game theoretic method depending on a simple sequence learning game played by multiple project agents and coordinated by a mediator agent. The minimization of *APD* and total makespan (*TMS*) were considered as the objectives. Zheng et al. [39] proposed a critical chain based elimination mechanism to resolve resource conflicts by considering both resource constraints and precedence constraints. The DMAS/EM algorithm was developed to obtain low *APD* of solutions even for large-size problems.

The above literature review shows that much more attention has been paid to solve the DRCMPSP with the objective of minimizing *APD*. Unfortunately, according to Vanhoucke et al. [17], most enterprises often faced the tardiness penalties as a result of project completion delays when they hiring subcontractors, research teams and maintenance crews. From a more realistic point of view, the senior manager would prefer to achieve a global *TTC* objective of multiple projects rather than a uniformity local delay goal of each project [40]. Since each project has different delay penalties, the multi-project schedule that minimizes *APD* does not ensure a minimized *TTC*. Each project manager is more concerned about completing the project as early as possible while the senior manager takes the *TTC* of the multi-project schedule as his primary consideration. Rather than aiming at the objective of minimizing *APD* as in the literature for DRCMPSP, this paper concentrates on developing a MAS-based approach to minimize *TTC*, which fulfills such gaps in existing works. In addition, a group of project managers plays sequential games to compete for global resources and eliminate conflicts. The sequential game theoretic approach has gained increasing popularity in optimizing resource allocation in grid environment [41,42] and integrating production and maintenance in the permutation flow shop sequencing problem [43]. We propose a two-stage decomposition approach to DRCMPSP by applying a new MAS and a sequential game-based negotiation mechanism.

## Problem description

In DRCMPSP, two types of renewable resources need to be distinguished which are required by each project. The local resources are only available to the corresponding project while the global resources are always shared by all projects. There is no relationship among projects except the shared global resources. Each individual project manager makes scheduling decision independently. The objective of the DRCMPSP proposed in this paper is to determine a multi-project schedule that minimizes *TTC*, such that the precedence constraints and local/global resource constraints are all satisfied. The symbols involved in this paper are defined as follows:

- There are *m* (*i* = 1, 2, …, *m*) projects to be scheduled simultaneously. Each project *i* has an arrival date *ad*_*i*_≥0 representing the earliest start time [44]. For each project *i*, the unit tardiness cost *tc*_*i*_≥0 is given [45].
- Each project *i* consists of *J*_*i*_ (*j* = 1, 2, …, *J*_*i*_) non-preemptable activities. *a*_*ij*_ stands for the *j*th activity of project *i*. Two dummy activities *a*_*i*0_ and $a_{i(J_{i}+1)}$ are added to each project *i* to represent the start and end time of the project, respectively. Each *a*_*ij*_ has a deterministic duration *d*_*ij*_.
- There are *s* (*g* = 1, 2, …, *s*) kinds of global resources shared by *m* projects. Global resource *g* is available with constant capacity *RR*_*g*_. In addition, there are *k* (*l* = 1, 2, …, *k*) types of local resources which only occupied by project *i*. Each local resource *l* has a constant capacity of *R*_*il*_. Every *a*_*ij*_ requires $r_{ij}^{l}$ units of local resources *l* and ${rr}_{ij}^{g}$ units of global resources *g* during its whole duration *d*_*ij*_. Without loss of generality, the durations and local/global resource requirements of the two dummy activities in each project are zero. The resources involved in this study are renewable resources that are not consumed as the progress of project.
- *E*_*ij*_ refers to the predecessor activity set of *a*_*ij*_. Only when all the activities in *E*_*ij*_ have been completed can *a*_*ij*_ be started.
- *T* denotes the whole planning horizon. It is the upper bound of all projects completion time. *t* (*t* = 0, 1, …, *T*) is the time point in the process of scheduling.

In addition, the final solution for the proposed DRCMPSP is represented by the vector *S* = (*S*_1_, *S*_2_,…, *S*_*m*_), where *S*_*i*_ = (*s*_*i*0_, *s*_*i*1_,…,$s_{i(J_{i}+1)}$) denotes the schedule of project *i* and *s*_*ij*_ is the start time of activity *a*_*ij*_.

Therefore, the total tardiness cost (*TTC*) is defined as below to evaluate the sequential game results for global resources coordination.

$$TTC(S)=\sum_{i=1}^{m}tc_{i}\cdot (s_{i(J_{i}+1)}-ad_{i}-CPL_{i})$$ (1)

where ($s_{i(J_{i}+1)}$-*ad*_*i*_) denotes the final makespan of project *i* and *CPL*_*i*_ stands for the critical path length of project *i* [5,12]. Therefore, *tc*_*i*_ ⋅ ($s_{i(J_{i}+1)}$-*ad*_*i*_-*CPL*_*i*_) is the tardiness cost incurred as the delay penalty of project *i*.

## Agent-based two-stage decomposition approach

### Multi-agent system for distributed decision process

**Fig 1:**
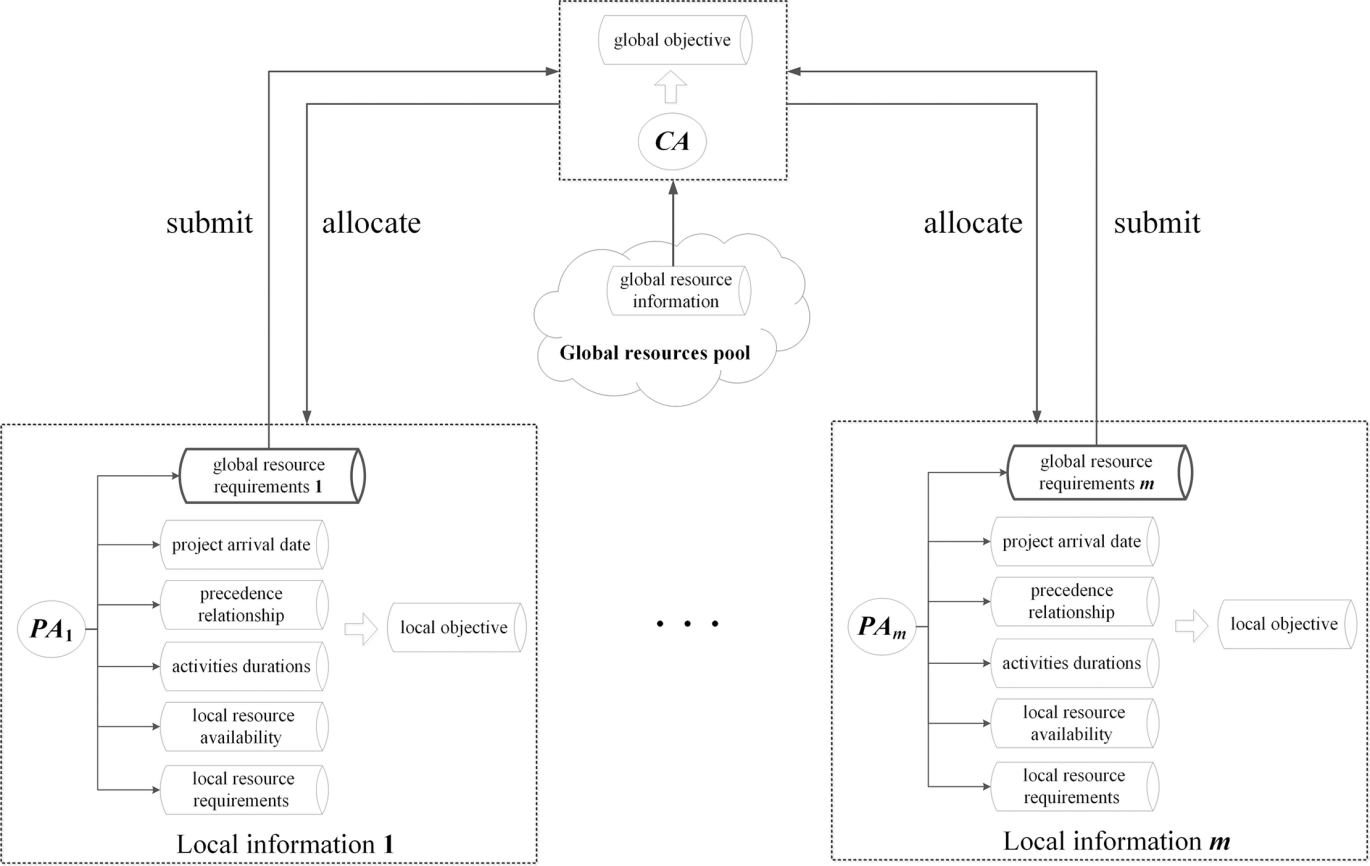
Multi-agent information and communication.

As demonstrated in Fig 1, the MAS consists of two types of self-interested and autonomous agents namely: Project Agent (*PA*) and Coordinator Agent (*CA*). If there is competition for shared resources among *PA*s, they are coordinated by *CA* to achieve the global objective of multi-project scheduling.

1. Project Agent (*PA*): Each *PA* is an independent local decision maker who plays the role of project manager in the company. There are as many *PAs* as projects. A *PA* is in charge of his own local information and responsible for generating a satisfactory initial local schedule privately. In addition, the *PAs* compete for global resources at each conflicting time slot and then modify their local schedule dynamically according to the coordination message received from *CA*. All the local information and scheduling scheme of individual *PAs* are secret to each other.
2. Coordinator Agent (*CA*): *CA* is a global decision maker who acts as the senior manager in the company. He holds the public information about the global objective and global resource availabilities. *CA* is mainly responsible for identifying the conflicting time slots, coordinating *PAs* to eliminate conflicts, and allocating global resources to related *PAs*.

In addition, all global resources are stored in the global resources pool. It is an important part in the MAS and opens for *CA* to offer global resources to each *PA*.

### Local initial decision-making stage

In stage one, owing to all the *PAs* do not know any information about the global resources allocation, each project is supposed to be scheduled independently with all the capacities of global resources. Since each *PA* is autonomous and self-interested, it will make a feasible and possibly optimal local schedule to achieve his own objective in this stage before *CA* identifies whether there are shared resource conflicts if all projects are scheduled simultaneously.

#### Local decision model

Each *PA* aims at generating a local schedule to complete his project as soon as possible. The local decision model for each project *i* is formulated as below:

$$min\;\sum_{t=0}^{T}t\cdot x_{i,J_{i}+1,t}$$ (2)

$$s.t.\;\sum_{t=0}^{T}x_{ijt}=1,\;\forall j=0,1,\ldots ,J_{i}+1$$ (3)

$$\sum_{t=0}^{T}\left(t‑d_{ij}\right)\cdot x_{ijt}-\sum_{t=0}^{T}t\cdot x_{iht}\geq 0,\;\forall (i,h)\in E_{ij}$$ (4)

$$\sum_{j\in V_{i}}\sum_{q=t}^{t+d_{ij}‑1}x_{ijq}\cdot r_{ij}^{l}\leq R_{il},\;\forall l=1,2,\ldots ,k,\;\forall t\in T$$ (5)

$$\sum_{j\in V_{i}}\sum_{q=t}^{t+d_{ij}‑1}x_{ijq}\cdot rr_{ij}^{g}\leq RR_{g},\;\forall g=1,2,\ldots ,s,\;\forall t\in T$$ (6)

$$\sum_{t=0}^{T}t\cdot x_{i,J_{i‑1}+1,t}\geq ad_{i}$$ (7)

$$x_{ijt}\in \{0,1\},\;\forall j=0,1,\ldots ,J_{i}+1,\;\forall t\in T$$ (8)

where *x*_*ijt*_ is a binary decision variable whose value is 1 if activity *a*_*ij*_ is completed at time *t*, otherwise 0.

Objective function (2) minimizes the project completion time, where element *J*_*i*_+1 is the subscript of last dummy activity used to record the completion time of project *i*. The project makespan is calculated by subtracting the arrival date from the project completion time [46]. Eq (3) assures that each activity is non-preemptive. Eq (4) is the precedence constraints. Constraints (5) and (6) limit the local and global resource requirements of all activities being executed at time *t* to the availabilities. Eq (7) forces the project to be started not earlier than its arrival date. The feasible domain constraints of decision variables are defined in Eq (8).

#### Local initial scheduling algorithm

Considering the good performance of genetic algorithm (GA) [29] and forward-backward scheduling (FBS) method [47,48] for RCPSP, we develop the FBHGA by combining the GA with FBS method to solve the local decision model for each PA. More concretely, the chromosome is encoded as an activity list and decoded by the forward-backward scheduling method with serial schedule generation scheme (SSGS). Firstly, the SSGS is used to forward schedule activities sequentially according to the activity list. The initial completion times of all activities and the initial project makespan are obtained by forward scheduling. Then, the completion time of last dummy activity is determined to start backward scheduling. Based on a priority list formed by sorting the activities’ completion times in descending order, activities are scheduled one by one at their latest reverse precedence and resource feasible start time. If the start time of the first dummy activity obtained in backward scheduling latter than the project arrival date, the project schedule can be improved by adjusting the start time of the dummy start node to equal 0 [48]. The completion times of all activities and the project makespan obtained in forward scheduling process are updates. Otherwise, the project schedule is determined as the forward schedule. We adopt the 2-tournament selection operator [49] and the two-point crossover operator [50], which perform better for RCPSP. Mutation is performed by exchanging two adjacent activities of selected chromosomes in turn with the precedence constraints. The algorithm is given below:

**Algorithm 1: FBHGA for *PA***_***i***_.

***Step 1*.** Generate initial population *POP*_*0*_.

***Step 2*.** Forward-backward scheduling.

(a) Forward schedule activities by SSGS.

(b) Record the completion times of all activities in descending order as priority list *L*.

(c) Backward schedule activities according to the priority list *L*.

(d) Record the start time of the first dummy activity as *s*_*i*0_.

    if *s*_*i*0_< *ad*_*i*_

        Determine the forward schedule as the initial schedule of project *i*;

    else

        *s*_*i*0_ ← *ad*_*i*_;

        Modify the backward schedule by shifting all the activities left *s*_*i*0_—*ad*_*i*_ units;

    end if

***Step 3*.** Genetic operation for population.

(a) Initialize generation counter *n* ← 0. *Gen* = generations.

(b) while *n*≤*Gen* do

            2-tournament selection operator;

            Two-point crossover operator;

            Mutation operator;

            Go to ***step 2***;

            *n* ←*n*+1;

        end while

***Step 4*.** Return the best schedule of project *i*.

### Global coordination decision-making stage

In the real world, each local initial schedule generated in stage one could be destroyed owing to the limited global resources. Therefore, it is indispensable to design an effective coordination mechanism for shared resources allocation without conflicts. In this section, the sequential game-based negotiation mechanism is proposed to eliminate the global resource conflicts among projects. Specifically, *CA* as a coordinator organizes sequential games for *PAs* when a shared resource conflict occurs. After several sequential game negotiations, *CA* determines the best subgame perfect Nash equilibrium according to the global decision model. Then multiple *PAs* resolve their local schedules with the allocated global resources from *CA* independently.

#### Sequential game-based negotiation mechanism

In game theory, the sequential game is defined as a game consisting of finite and at least two players where each player takes actions at different times or in turn. In other words, one player selects his action before other players choosing theirs, which means that players who make move later have additional information about the actions of those who make move before it in the game [51]. As a rational player, the toper one in the sequence has first-mover advantages since his global resources requirements can be satisfied firstly. In DRCMPSP, we regard the time as discrete resources, namely time slots of a resource. The conflicting time slot is a decision point where the global resource requirements exceed the available amounts. The sequential game among multiple projects is composed of five key elements.

- Players: At each resource conflicting time slot, the *PAs* who require the limited global resources to schedule their activities are treated as the sequential game players.
- Strategies: The feasible start time slots of conflicting activities at which no resource conflict occurs are defined as strategies or actions for *PAs*.
- Sequence: Every player takes action in an order. A player chooses its strategy depending on actions taken by other players ordered before him.
- Payoff: It represents the player’s preferences for chosen strategies. Since each *PA* aims at completing the project as soon as possible, the opposite of the project tardiness cost is evaluated as the payoff of associated *PA*.
- Equilibrium: The combined strategies selected sequential rationally by all the game players for their activities at a certain conflicting time point are described as the subgame perfect Nash equilibrium.

For a sequential game, the subgame is defined as a subset of the game that contains an initial node and all its successor nodes. Nash equilibrium describes the players’ behavior in a steady state, where any player has no incentive to change his/her own decision. A subgame perfect equilibrium is an equilibrium strategy profile that in no subgame can any player do better by taking an action different from the profile while every other player adheres to it [51], according to which the resource conflict is resolved. To obtain the subgame perfect equilibrium for a sequential game, the Nash equilibrium is defined for each subgame as below.

**Definition (Nash equilibrium).** Let *A* be an action profile including all feasible start time slots of conflicting activities for game players, in which the action set of each player *PA*_*i*_ is *A*_*i*_. Let $aa_{i}^{*}(\forall aa_{i}^{*}\in A_{i})$ and *aa*_*i*_ (∀*aa*_*i*_ ∈ *A*_*i*_) be the optimal and any feasible start time slots chosen by player *PA*_*i*_, respectively. Then ($aa_{i},aa_{‑i}^{*}$) represents the action profile in which all players other than *PA*_*i*_ adhere to choose the best action of $aa_{q}^{*}$ (∀*q* ∈ {1,…,*i* − 1,*i* + 1,…,*m*}) while *PA*_*i*_ chooses *aa*_*i*_. If $aa_{i}=aa_{i}^{*}$, then of course ($aa_{i},aa_{‑i}^{*}$) = ($aa_{i}^{*},aa_{‑i}^{*}$) = aa* ($aa^{*}=\left\{aa_{1}^{*},\ldots ,aa_{i}^{*},\ldots ,aa_{m}^{*}\right\}$). In a normal-form game, the action profile aa* is a Nash equilibrium if, for each player *PA*_*i*_ and any other feasible time slots actions *aa*_*i*_ ∈ *A*_*i*_, $aa_{i}^{*}$ is the best response of player *PA*_*i*_ to the strategies $aa_{‑i}^{*}$ specified for the other players. Equivalently, for each player *PA*_*i*_ and every feasible time slots action *aa*_*i*_ of player *PA*_*i*_,

$$u_{i}(aa^{*})\geq u_{i}(aa_{i},aa_{‑i}^{*})$$ (9)

where *u*_*i*_ is a payoff of player *PA*_*i*_.

In the light of the sequential structure, each player’s strategy need to be optimal in the sequential game to avoid a perturbed steady state or achieve the Nash equilibrium. The optimal strategy chosen by each PA is proposed and proved as follows.

**Proposition.** In a sequential game, the strategy best for any given player depends on the other players’ actions. The subgame perfect Nash equilibrium is determined if, every player is sequentially rational which means that no matter what happened in the past, each player *PA*_*i*_ prefers to choose the optimal strategy i.e. the earliest available time slots according to the decision-making sequence. At each conflicting time slot, the *PAs* involved in the sequential game will employ the virtual scheduling method (see Section “Resource conflicts dynamic elimination algorithm”) to resolve their respective local schedules.

**Proof.** Suppose that it’s time for *PA*_*i*_ to make decision for activity *a*_*ij*_, *PA*_*i*_ chooses a strategy other than the best one which is later than the earliest available time slots. Then *a*_*ij*_ is scheduled at the time slot determined by the chosen strategy. According to the virtual scheduling method, unscheduled activities of *PA*_*i*_ belonged to the successors of *a*_*ij*_ will not start earlier than its local initial start time obtained in stage one, which means the project makespan cannot be shortened. According to Eq (1), the tardiness cost of project *i* will not be reduced. In other words, no additional payoffs are gained for *PA*_*i*_. As a rational project agent, no changes will be made to realize his local objective and a steady state is achieved. Therefore, in each iteration of sequential game negotiation, the strategy combination that every player chooses the earliest available time slots orderly is consist of subgame perfect Nash equilibrium.

#### Global decision model

For each conflicting time slot, *CA*, acts as a senior manager, is responsible for finding a best sequence with the best subgame perfect Nash equilibrium to meet the following global decision model:

$$\underset{\pi \in \Pi }{min}\;TTC(S_{v}(\pi ,t_{c}))$$ (10)

where objective function (10) seeks to minimize the *TTC* of the multi-project scheduling *S*_*v*_ (*π*, *t*_*c*_) obtained by using a virtual scheduling method (see Section “Resource conflicts dynamic elimination algorithm”) at the conflicting time slot *t*_*c*_ following a sequence *π* ∈ Π. It is worth noting that the global resource feasibility at *t*_*c*_ is guaranteed by the virtual scheduling method.

#### Resource conflicts dynamic elimination algorithm

**Fig 2:**
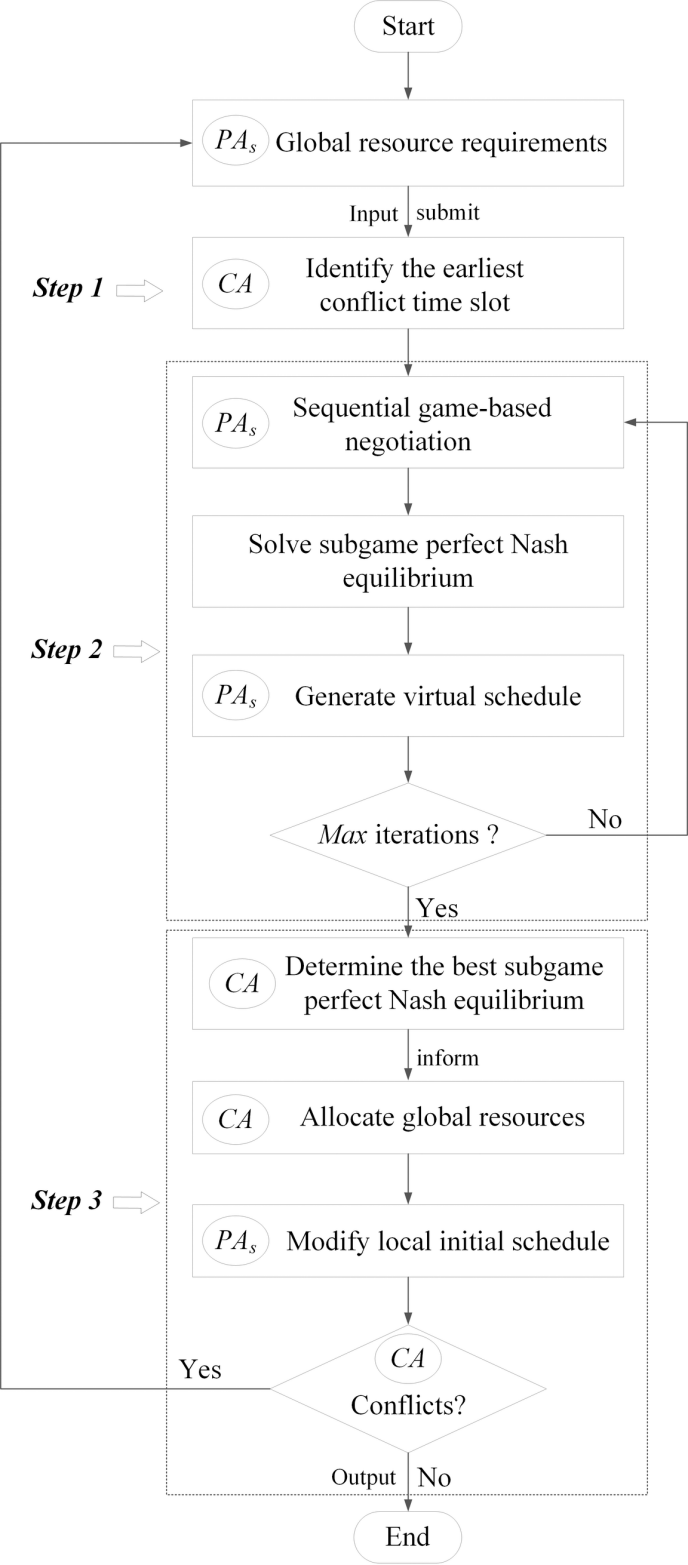
Flowchart of conflicts elimination.

After all the *PAs* generating their local initial schedules in stage one, a local activity list of each *PA* is generated by ordering activities with ascending start times. As shown in Fig 2, the information of global resource requirements of *PAs* is submitted to *CA*. Afterwards, *CA* identifies the resources conflicts and eliminates them one by one. For a sequential game involving *N* players, there will be a total *N*! of possible sequences of players, which is well-known to be NP-hard. Therefore, a randomized search heuristic method is used to determine the best sequence and best subgame perfect Nash equilibrium. More concretely, at the first conflicting time slot, *CA* allocates global resources to the corresponding projects by initiating multiple iterations of sequential game-based negotiation. In each iteration, the sequence of game players is determined randomly. Then the projects involved in the sequential game are virtually scheduled by a given method. Specifically, for each *PA*, activities starting earlier than the conflicting time are virtually scheduled as its local initial schedule, activities starting at the conflicting time are virtually scheduled according to the subgame perfect Nash equilibrium, and activities starting later than the conflicting time are virtually scheduled by a fast heuristic procedure with SSGS based on the local activity list determined in stage one. After several iterations of negotiation, *CA* determines the best subgame perfect Nash equilibrium with minimal *TTC* according to the global decision model to eliminate the current resource conflict. Followed by *CA* allocating global resources, each of the corresponding *PA* modifies its local schedule by the above method. The following conflicts are identified and solved in the same way. Until there is no conflicts at all time slots, the whole process is terminated. Details of the conflicts dynamic elimination process are described as Algorithm 2.

Algorithm 2: Resources conflicts dynamic elimination.

Initialize: current time *t* ← 0.

*Step 1*. Identify conflicting time slots.

    while *t*≤*T* do

        Each *PA* submits its global resource requirements information to *CA*;

        Determine the earliest conflicting time slot *t*_*c*_;

        Go to *step 2*;

    end while

*Step 2*. Perform sequential game-based negotiation on *PAs*.

(a) Determine the set of game players *GP*.

(b) Initialize negotiation iteration *N* ← 0, Max iterations←Ω.

(c) Virtually schedule each local project.

    while *N*≤Ω do

        Determine a game sequence;

        Solve subgame perfect Nash equilibrium;

    for each *PA*_*i*_ in *GP* do

        Activities with start time *s*_*ij*_<*t*_*c*_ are virtually scheduled as its local initial schedule;

        Activities with start time *s*_*ij*_ = *t*_*c*_ are virtually scheduled according to the subgame perfect Nash equilibrium;

        Activities with start time *s*_*ij*_>*t*_*c*_ are virtually scheduled by SSGS based on the local initial activity list;

    end for

        Compute *TTC*_*N*_ according to Eq (1);

        *N* ←*N* +1;

    end while

*Step 3*. Coordinate global resources allocation.

(a) Determine the best subgame perfect Nash equilibrium with minimal *TTC*.

(b) *CA* allocates the global resources to corresponding *PAs*.

(c) Each *PA* modifies and updates its local initial scheduling.

(d) *t* ←*t* +1;

    Return to *step 1*.

Output: final multi-project schedule and final *TTC*.

## An illustrative example

**Fig 3:**
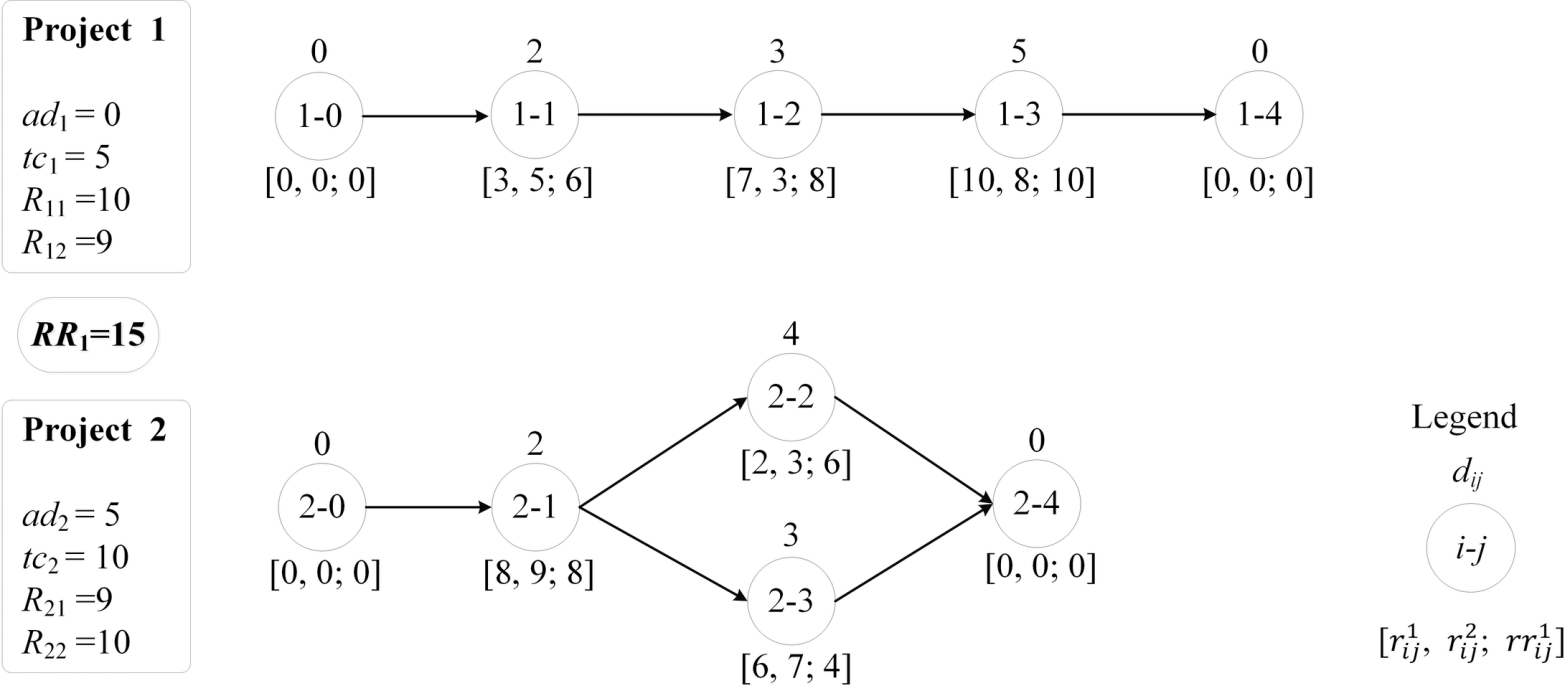
Information for illustrative example.

In this section, we present a simple example to demonstrate the proposed approach for DRCMPSP. In addition, we use this example to illustrate the fact that the multi-project schedule that minimizes *APD* cannot guarantee a minimum *TTC* for the senior manager. An illustrative multi-project scheduling problem with two projects is considered. Each project consists of five activities where two of them are dummies. Each activity requires $r_{ij}^{1},\;r_{ij}^{2}$ units of local resources and ${rr}_{ij}^{1}$ units of global resources. Fig 3 demonstrates the activity-on-node (AON) networks and all information of the two projects. The duration *d*_*ij*_ and requirements of local/global resources $r_{ij}^{1},\;r_{ij}^{2}$ and ${rr}_{ij}^{1}$ of each activity are marked on the top and bottom of the corresponding node, respectively. Dummy activities have zero duration and resources requirements. Other information about arrival date *ad*_*i*_, unit tardiness cost *tc*_*i*_, and local resource capacities *R*_*il*_ of each project is presented in the box left to the corresponding project AON network. As shown between the two boxes, the capacity *RR*_1_ of global resource is assumed to be 15.

In the following, we elaborate the whole two-stage decision-making process.

*Stage one*: Local initial decision-making process

**Fig 4:**
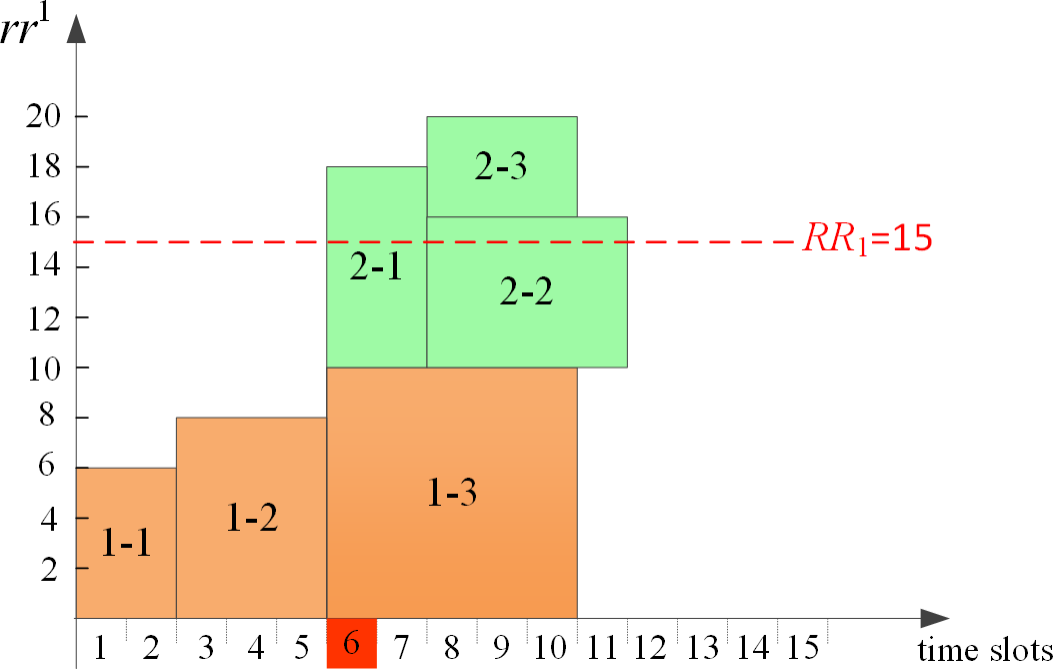
Initial schedule of each project.

As shown in Fig 4, each *PA* generates an initial schedule independently to minimize the project completion time. It can be seen that the makespan of project 1 and project 2 are 10 and 6 respectively, which are equal to the critical path length (*CPL*_*i*_) of each corresponding project. Obviously, the global resource requirements violate the given capacity from time slot 6. Therefore, it is necessary for *CA* to eliminate the global resource conflicts.

*Stage two*: Global coordination decision-making process

**Fig 5:**
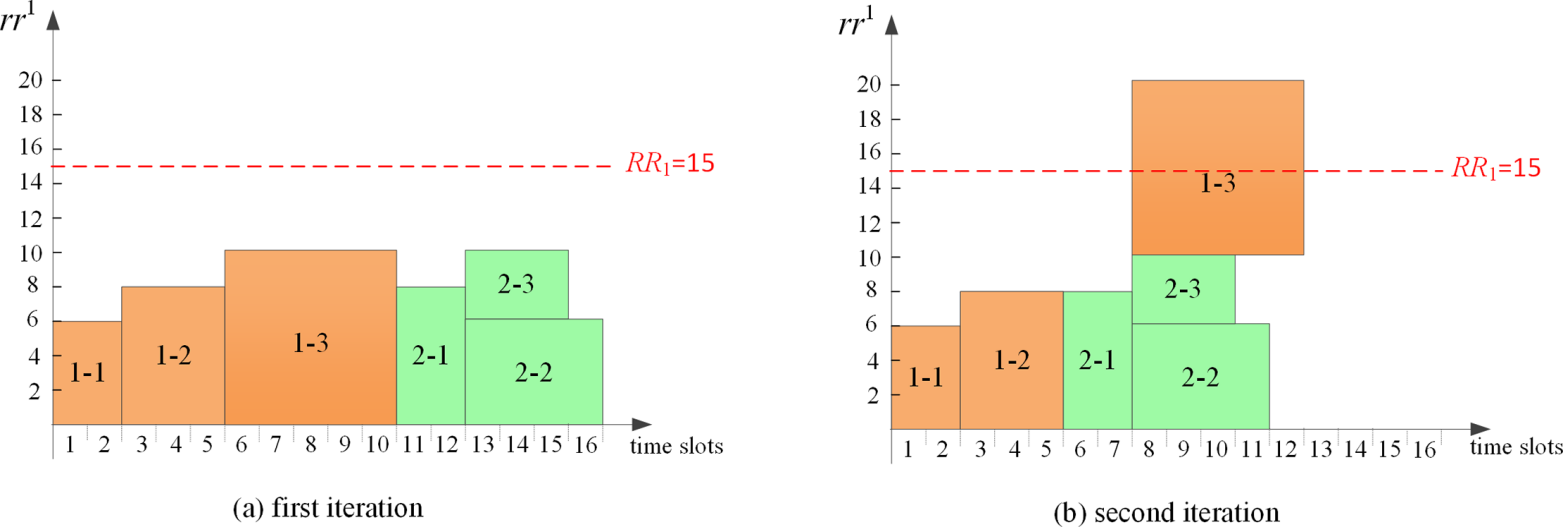
Virtual schedule of multi-project after eliminating the first conflict at time slot 6.

**Fig 6:**
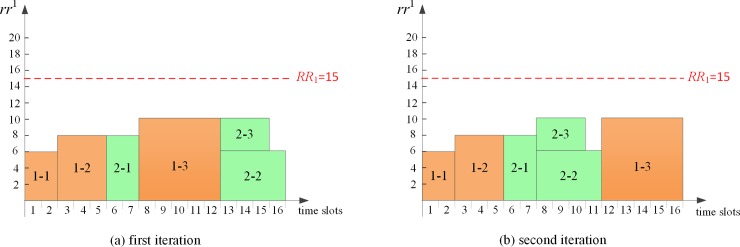
Virtual schedule of multi-project after eliminating the second conflict at time slot 8.

After each *PA* produces its local initial schedule without consideration of sharing global resources with other *PAs*, *CA* receives the global resource requirements information from each *PA*. The first conflicting time slot 6 is identified firstly by *CA*, and then the process of sequential game-based negotiation between *PA*_*1*_ and *PA*_*2*_ begins. At the first conflicting time slot 6, game players *PA*_*1*_ and *PA*_*2*_ decide when to start *a*_13_ and *a*_21_, respectively. Time slots after time point 5 constitute the strategies for each *PA* to execute its corresponding activities. Suppose the game sequence is {*PA*_*1*_, *PA*_*2*_} in the first iteration, the subgame perfect Nash equilibrium is determined, i.e. *a*_13_ and *a*_21_ are started at time slot 6 and time slot 11 by *PA*_*1*_ and *PA*_*2*_, respectively. It can be calculated that the *TTC* of the virtual multi-project schedule in the first iteration is 50. In the same way, if the game sequence is {*PA*_*2*_, *PA*_*1*_} in the second iteration, the subgame perfect Nash equilibrium is determined, i.e. *PA*_*1*_ schedules *a*_13_ at time slot 8 and *PA*_*2*_ schedules *a*_21_ at time slot 6. The *TTC* of virtual multi-project schedule in the second iteration is 10. The virtual multi-project schedule in each iteration is shown in Fig 5. Then *CA* determines the best subgame perfect Nash equilibrium with the objective of minimizing *TTC*. Clearly, *CA* prefers to allocate global resources according to the negotiation result in the second iteration (Fig 5(B)). Similarly, the virtual schedule in each iteration after eliminating the second conflict is demonstrated in Fig 6. *CA* will choose the schedule in Fig 6(B) as its final decision since the *TTC* value is smaller.

Since the unit delay penalty may differ by projects, a multi-project schedule with minimized *APD* is not aligned with the objective of *TTC* minimization. As illustrated in Fig 5(A), the *APD* in this case is minimized with the value of 2.5 while the *TTC* is 50. In Fig 6(B), the schedule is determined with the minimum *TTC* of 30 while the *APD* is 3. Therefore, our study focus on minimizing the *TTC* objective of multi-project from the portfolio perspective rather than devoting to obtain lower *APD* on the project level.

## Computational study

In order to evaluate the performance of the proposed approach, we carry out a series of computational experiments. Algorithms are coded in Matlab R2013a. All the experiments are executed on a single computer with Inter Core i7 (3.4GHz, 8GB RAM). The performance of the solutions is evaluated in two aspects:

- The local initial schedule obtained in stage one are evaluated according to project makespan (*MS*_*i*_). The results are also compared with the optimal solutions obtained by the B&B algorithm to demonstrate the quality of the schedules obtained by using FBHGA.
- The performance of the proposed sequential game-based negotiation mechanism is evaluated based on the measure of *TTC*. We conduct the pre-experiment by using different numbers of negotiation iterations. We also compare the performance of our algorithm in terms of *TTC* with two state-of-the-art variants of the coordination mechanism integrated in a new multi-agent system (CMAS) based decentralized methods: CMAS/ES with the (μ,λ)-coordination mechanism [18], and CMAS/SA with the mechanism proposed by Fink [52]. Since these two existing methods focus on minimizing *APD*, we use the feasible multi-project schedules produced by the two methods which are available from MPSPLIB (http://www.mpsplib.com, last check of address: 16 November 2017) to compute the *TTC* of each multi-project instance according to Eq (1) for comparison.

### Problem instances

The proposed algorithm is tested on all the 140 problem instances from MPSPLIB (Supporting Information file S1 Appendix). As shown in Table 1, these instances are classified into 20 subsets according to the Utilization Factors (*UF*). Each problem subset is named as “MP*J*_*i*_*_m*” (MP subsets) or “MP*J*_*i*_*_m*AC” (MPAC subsets), where the number of activities *J*_*i*_ per project is 30, 90, or 120, and the number of projects *m* is 2, 5, 10, or 20. Each problem instance is provided with *s* and *k* types of global and local resources, respectively. *UF*_*g*_ is the utilization factor of global resource *g*∈*G* indicating the ratio of the total requirements *rr*^*g*^ to the constant capacity *RR*_*g*_ available in each time period over the global critical path [18]. *UF* is calculated as Eq (11) denoting the maximum tightness of the constraints on required global resources [53].

**Table 1:** Problem instances for DRCMPSP (Homberger 2012).

Problem subset	*NOI*	Characterization per instance				*UF*_*AV*_
		*m*	*J*_*i*_	Problem size	(*s*; *k*)	
MP30_2	5	2	30	60	(1; 3)、(2; 2)、(3; 1)	0.84
MP90_2	5	2	90	180	(1; 3)、(2; 2)、(3; 1)	0.57
MP120_2	5	2	120	240	(1; 3)、(2; 2)、(3; 1)	1.31
MP30_5	5	5	30	150	(1; 3)、(2; 2)、(3; 1)	0.82
MP90_5	5	5	90	450	(1; 3)、(2; 2)、(3; 1)	0.61
MP120_5	5	5	120	600	(1; 3)、(2; 2)、(3; 1)	1.32
MP30_10	5	10	30	300	(1; 3)、(2; 2)、(3; 1)	2.38
MP90_10	5	10	90	900	(1; 3)、(2; 2)、(3; 1)	1.14
MP120_10	5	10	120	1200	(1; 3)、(2; 2)、(3; 1)	1.91
MP30_20	5	20	30	600	(1; 3)、(2; 2)、(3; 1)	3.37
MP90_20	5	20	90	1800	(1; 3)、(2; 2)、(3; 1)	0.90
MP120_20	5	20	120	2400	(1; 3)、(2; 2)、(3; 1)	0.87
MP90_2AC	10	2	90	180	(4; 0)	2.27
MP120_2AC	10	2	120	240	(4; 0)	1.36
MP90_5AC	10	5	90	450	(4; 0)	4.99
MP120_5AC	10	5	120	600	(4; 0)	3.80
MP90_10AC	10	10	90	900	(4; 0)	3.85
MP120_10AC	10	10	120	1200	(4; 0)	2.61
MP90_20AC	10	20	90	1800	(4; 0)	2.70
MP120_20AC	10	20	120	2400	(4; 0)	3.65

*NOI* no. of instances; *m* no. of projects; *J*_*i*_ no. of activities of project *i*; *s* no. of global resource types; *k* no. of local resource types of each project *i*; *UF*_*AV*_ average utilization factor.

$$UF=\underset{g\in G}{max}\;UF_{g}$$ (11)

The *UF*_*AV*_ in Table 1 is the average of the *UF* values of a given problem subset. *UF* >1 indicates a medium to high overload which means the global resource conflicts among multiple projects are relatively strong, while *UF* <1 denotes a low to medium overload which means the conflicts are relatively weak [12].

### Results and analysis

The performance of the proposed two-stage scheduling algorithm for DRCMPSP is evaluated on two stages separately. For algorithm 1, the best parameter settings of GA are determined by pre-experiments as follows: the population size *POP*_*0*_ = 60, the number of generations *Gen* = 100, the crossover and mutation rates are 0.9 and 0.1 respectively. For algorithm 2, to obtain the final solution in reasonable CPU running time, we set the number of negotiation iterations Ω to three levels: 1, 5, and 10, respectively.

#### Comparison of local initial schedule

To compare the results in terms of project makespan of each local initial schedule obtained in stage one, we have performed the exact B&B algorithm and the proposed FBHGA on 1295 single projects from the 140 multi-project problem instances. The optimal solutions of some small-size single project instances are obtained by the B&B algorithm embedded in RESCON software [19]. Note that the solvable solutions obtained by RESCON are all optimal results and any solutions cannot be found if the RESCON runs out of memory.

Table 2 shows the comparison of the average project makespan (*APM*), the number of projects with optimal solutions (*NPOS*), and the number of projects with no feasible solutions (*NPNS*) solved by B&B and FBHGA respectively, meanwhile the last column shows the average relative deviation (*ARD*) of the non-optimal solutions obtained by FBHGA from the optimal results reached by B&B of each subset. It is clear that out of the 1295 single projects, B&B can obtain optimal solutions for 954 projects and 89.3% of which are also obtained by FBHGA. In addition, the more the activities involved in a project, the less the possibility that a feasible solution can be obtained by B&B before running out of memory, especially for projects with 120 activities. However, the FBHGA can achieve a feasible scheduling solution efficiently for any size project case. For each problem subset, the results of *APM* obtained by FBHGA and B&B are very close with respect to the solvable solutions. Furthermore, the *ARD* of each subset is lower than 5%. The average of *ARD* over all of the 20 subsets is less than 1% (0.81%). According to the statistical analysis of T test, there is no significant difference between B&B and FBHGA solutions (the *P*_value is 0.750). It fully turned out that the FBHGA performs well on generating high-quality local initial schedules with satisfactory makespan for every *PA* in stage one.

**Table 2:** Comparison of results obtained by B&B and FBHGA in stage one.

Problem subset	*NSP*	B&B			FBHGA			*ARD* (%)
		*APM*	*NPOS*	*NPNS*	*APM*	*NPOS*	*NPNS*	
MP30_2	10	54.30	10	0	55.30	4	0	1.83
MP90_2	10	93.50	8	2	93.50	8	0	0
MP120_2	10	98.00	1	9	102.00	0	0	4.08
MP30_5	25	58.80	25	0	59.72	18	0	1.50
MP90_5	25	88.85	20	5	88.90	19	0	0.04
MP120_5	25	101.75	4	21	102.00	3	0	0.30
MP30_10	50	52.26	50	0	53.04	32	0	1.55
MP90_10	50	88.19	37	13	88.19	37	0	0
MP120_10	50	103.57	14	36	104.50	8	0	0.94
MP30_20	100	57.09	100	0	57.59	76	0	0.84
MP90_20	100	92.59	88	12	93.07	76	0	0.49
MP120_20	100	103.53	15	85	104.47	10	0	0.90
MP90_2AC	20	76.50	4	16	76.50	4	0	0
MP120_2AC	20	85.50	12	8	86.58	8	0	1.38
MP90_5AC	50	83.67	6	44	84.67	3	0	1.22
MP120_5AC	50	88.73	15	35	89.27	12	0	0.64
MP90_10AC	100	78.97	70	30	79.26	66	0	0.32
MP120_10AC	100	94.62	86	14	94.67	84	0	0.05
MP90_20AC	200	79.06	200	0	79.06	200	0	0
MP120_20AC	200	97.21	189	11	97.29	184	0	0.09
Total	1295	83.83^a^	954	341	84.48^a^	852	0	0.81^a^

*NSP* no. of projects; *APM* average project makespan of the problem subset; *NPOS* no. of projects with optimal solutions; *NPNS* no. of projects with no feasible solutions; *ARD* (%) average relative deviation

^a^ Average of column values over all problem subsets.

#### Performance analysis of coordination mechanism

**Fig 7:**
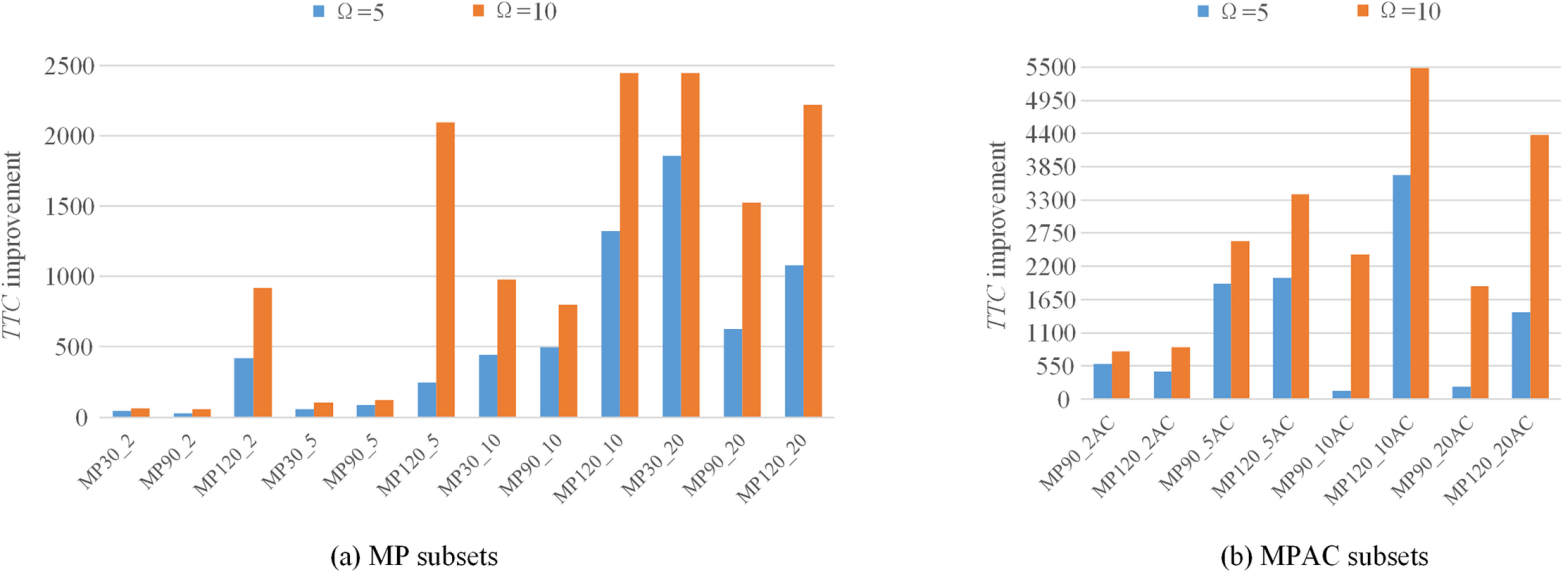
Comparison of *TTC* improvements of MP subsets and MPAC subsets.

**Fig 8:**
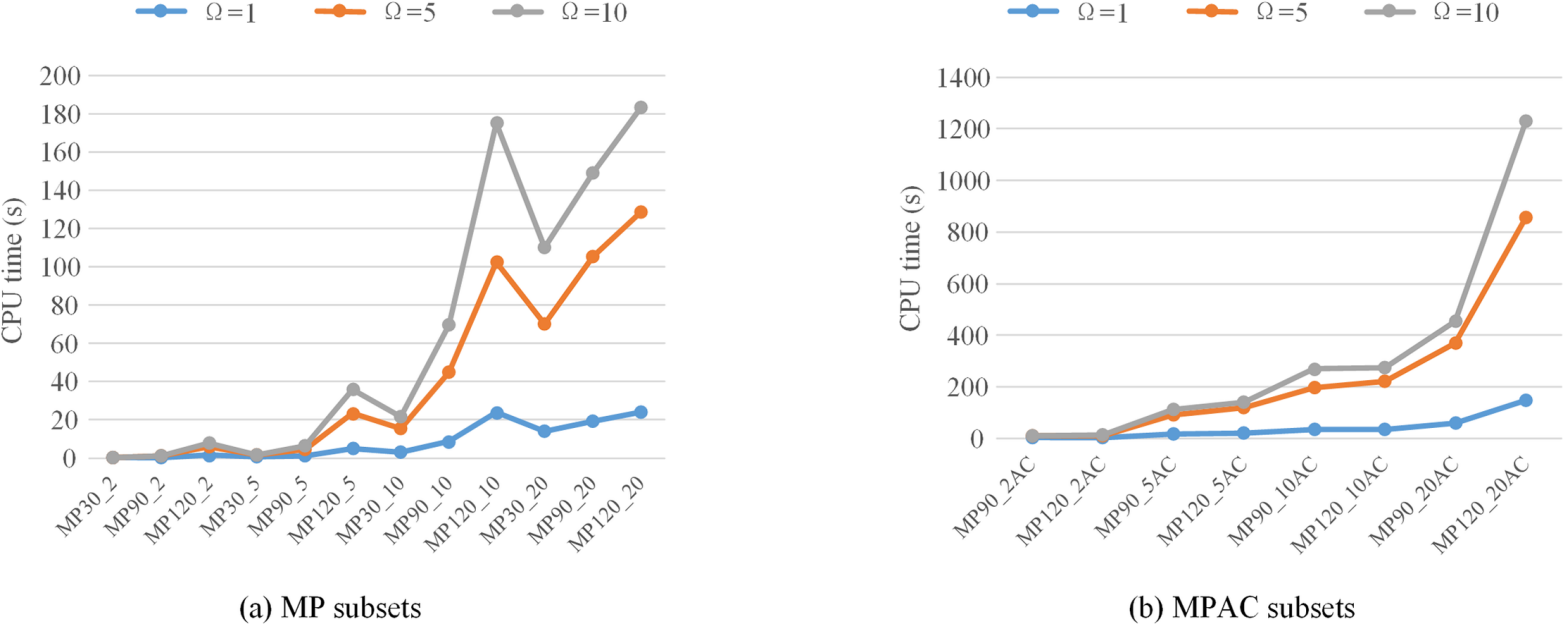
Comparison of CPU time of MP subsets and MPAC subsets.

It should be noted that the number of iterations Ω for sequential game negotiation is considered in three modes i.e. 1, 5, and 10. As revealed in Table 3, with the increasing of iterations, the average *TTC* of each subset reduces while the CPU running time increases. The effect of the number of iterations on either *TTC* or CPU running time is statistically significant based on the related-samples Kendall’s coefficient of concordance test (the *P*_value of each test is 0.000). The reason is that the more the negotiation iterations, the greater the possibility of finding better solutions and thus the more CPU time is consumed. Compared to the results with Ω = 1, the improvements on *TTC* of MP subsets and MPAC subsets with Ω = 5 and Ω = 10 are depicted respectively in Fig 7. Clearly, the *TTC* results with Ω = 10 are improved more than that with Ω = 5 by comparing the corresponding results with Ω = 1 for all subsets. Additionally, the greater improvements with both Ω = 5 and Ω = 10 are made for large-size subsets with high *UF*_*AV*_. Fig 8 illustrates the CPU time results of MP subsets and MPAC subsets with different sets of iterations. It is clear that the larger the size of subsets, the longer the CPU running time is. Meanwhile, the MPAC subsets generally run longer than the MP subsets with the fact that more conflicts need to be solved for MPAC subsets as a result of the higher *UF*_*AV*_. Since the better solution of each multi-project instance can be obtained within about 20 min, we set Ω = 10 for following study analysis.

**Table 3:** Computational results with different number of negotiation iterations.

Problem subset	Ω = 1		Ω = 5		Ω = 10	
	*TTC*	CPU (s)	*TTC*	CPU (s)	*TTC*	CPU (s)
MP30_2	474	0.12	429.2	0.43	412.6	0.54
MP90_2	949.2	0.40	924	1.19	890.4	1.21
MP120_2	12688.6	1.50	12266.6	6.08	11767.8	7.95
MP30_5	1592.4	0.85	1535.8	1.57	1486.4	1.62
MP90_5	2952.4	1.42	2866.2	4.53	2827.4	6.41
MP120_5	23300	5.10	23052	23.37	21205	35.74
MP30_10	10438.8	2.97	9997.2	15.58	9461	21.65
MP90_10	19678.2	8.59	19180.4	44.88	18879	69.72
MP120_10	78803.8	23.79	77484.2	102.65	76361.2	175.23
MP30_20	61415.6	14.02	59556.6	70.35	58972.6	110.11
MP90_20	33573.6	19.41	32943.4	105.45	32046.8	149.08
MP120_20	44071.8	24.09	42992	128.86	41850.4	183.34
MP90_2AC	11186.2	2.51	10601.4	10.00	10394	10.19
MP120_2AC	7656.4	2.77	7193.9	11.33	6796.7	13.38
MP90_5AC	59236.4	17.75	57322	91.19	56614.9	112.19
MP120_5AC	114389.1	20.79	112376.3	118.73	110993.3	140.02
MP90_10AC	103492.5	33.97	103358.7	196.51	101098.8	269.80
MP120_10AC	123481.7	35.89	119767.8	221.88	117991.9	276.27
MP90_20AC	79310.7	60.99	79099.9	369.63	77437.4	453.42
MP120_20AC	424001.6	146.93	422560.2	856.94	419621.6	1230.46

The *TTC* and CPU (s) are the average values of each problem subset.

Given the complexity and diversity of the practical multi-project scheduling environment, senior managers in the company often have to deal with multiple projects under different degrees of conflicts [54]. To evaluate the performance of the proposed sequential game-based negotiation mechanism on different instance subsets with various problem sizes and degrees of conflicts, we analyze two indicators: *DTTC* and NC. *DTTC* is the deviation of the final actual total tardiness cost (*TTC*(*S*), obtained in stage two) from the initial total tardiness cost (*TTC*(*S*_*initial*_), obtained in stage one). NC represents the number of conflicts occurred in the whole multi-project scheduling horizon.

**Fig 9:**
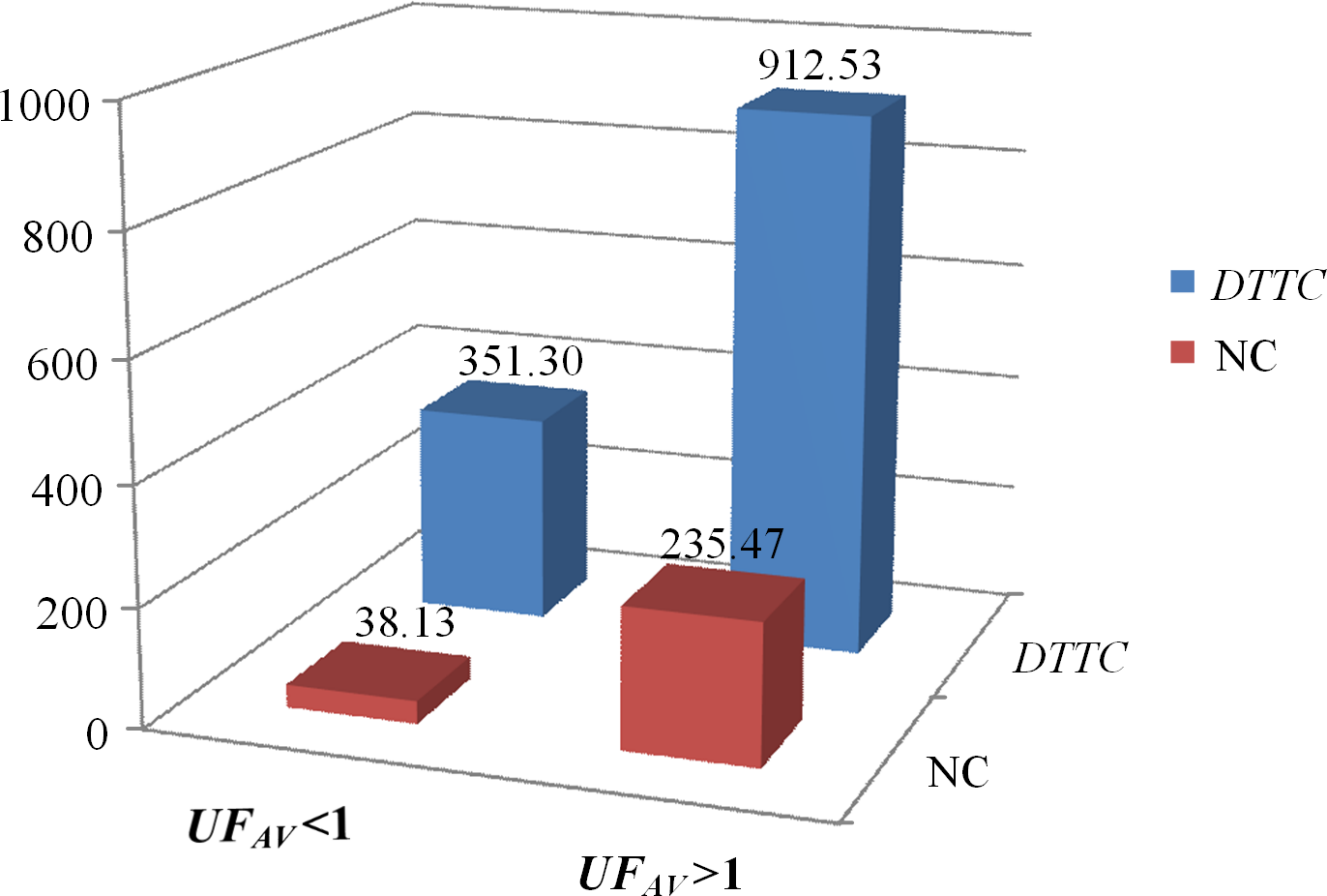
Comparisons of *DTTC* and NC under different degrees of conflict.

According to the values of *UF*_*AV*_, the 20 problem subsets are classified into two groups: subset group with *UF*_*AV*_ <1 and the other one with *UF*_*AV*_ >1. As shown in Fig 9, the average values of *DTTC* and NC of the subset group with *UF*_*AV*_ <1 are significantly lower than those values of the subset groups with *UF*_*AV*_ >1. It reveals that the stronger the degrees of conflicts among multiple projects, the greater the final actual *TTC* value deviates from the initial *TTC* value, and the more the number of conflicts existing in the process of multi-project scheduling. The results indicate that the multi-project scheduling environment will become complicated and more *TTC* will be incurred when the global resource conflicts among projects are relatively strong.

**Fig 10:**
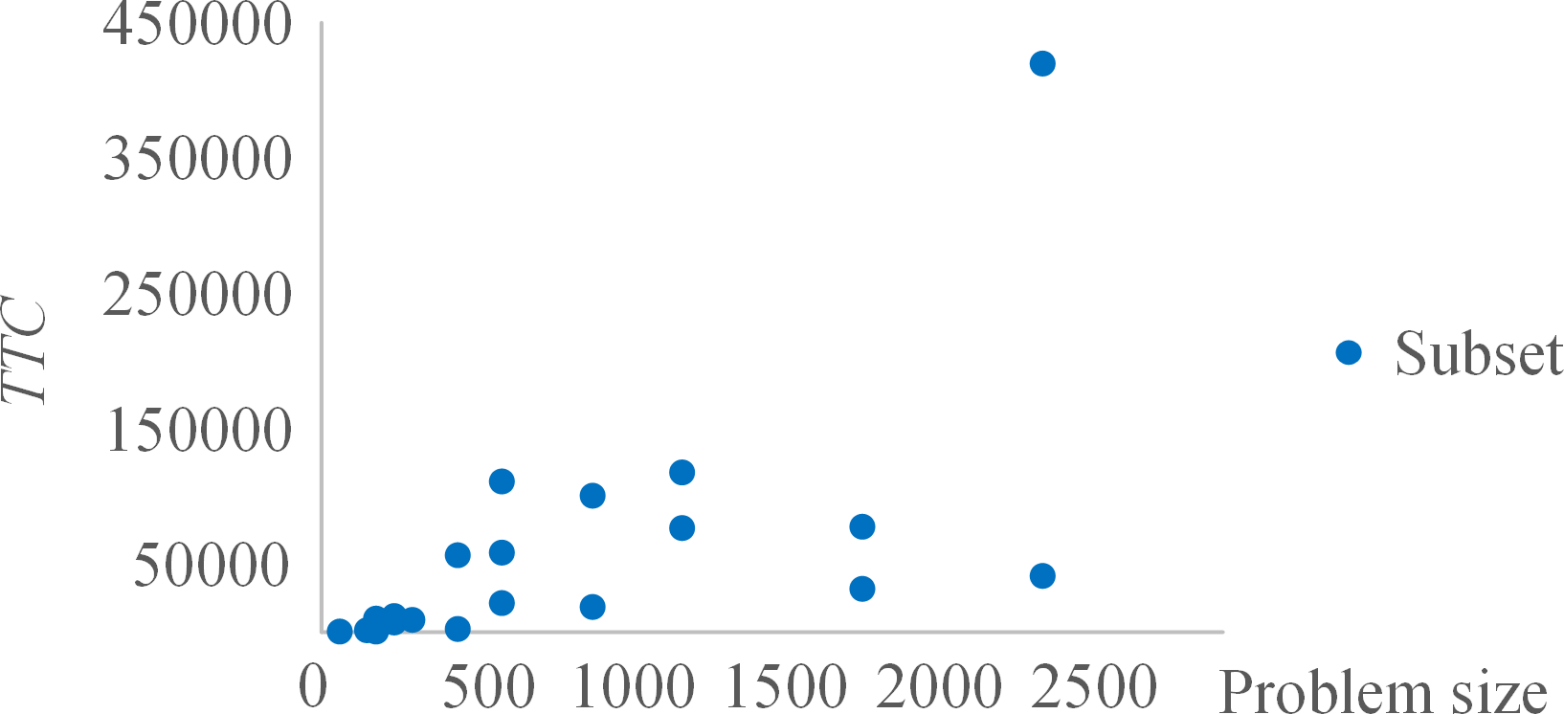
Relationship between problem size and *TTC* result.

Fig 10 illustrates the relationship between problem size and *TTC* result for all subsets. According to the one-way ANOVA test, the problem size has no significant effect on *TTC* (the *P*_value is 0.558), which is mainly because the unit tardiness cost of each project in a multi-project instance is different.

**Fig 11:**
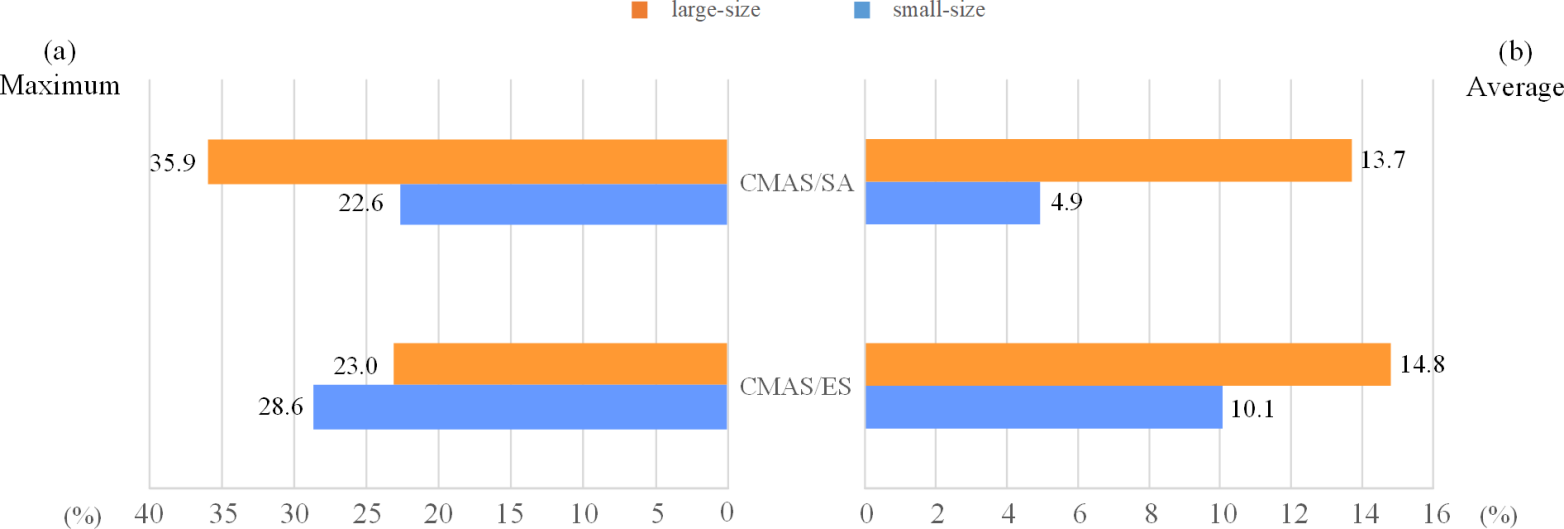
Percentage improvement with respect to different problem sizes.

**Fig 12:**
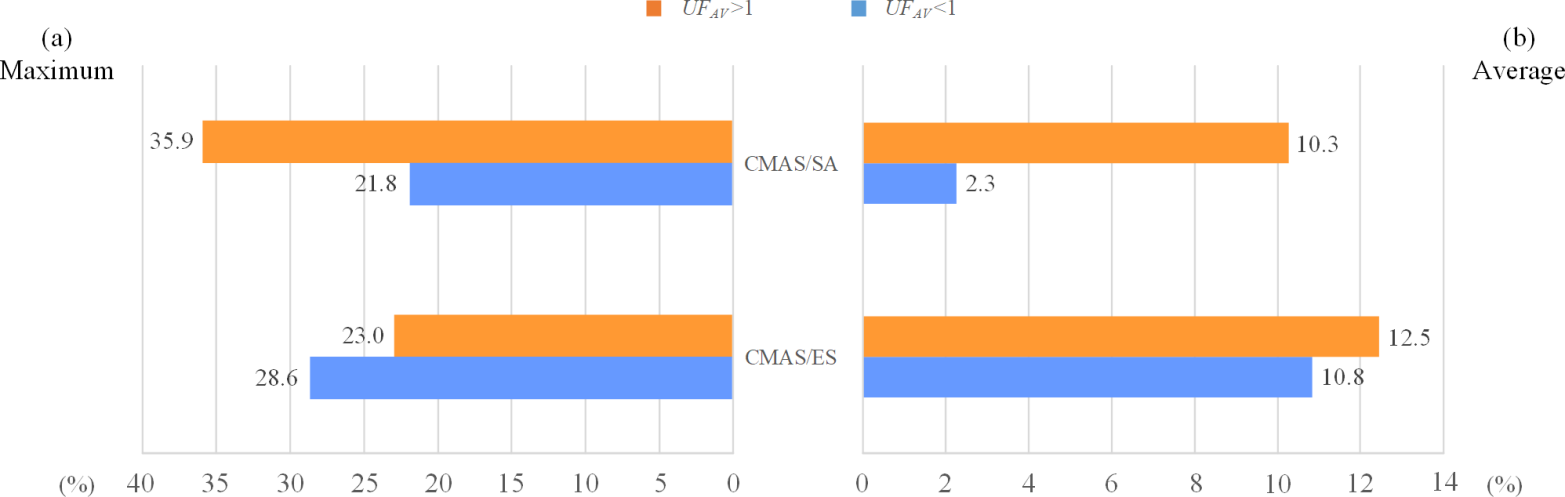
Percentage improvement with respect to different degrees of conflicts.

In order to evaluate the performance of the proposed two-stage decomposition approach with respect to *TTC*, the comparisons with two existing decentralized methods i.e. CMAS/ES and CMAS/SA are shown in Table 4. The best results of each subset are marked in bold, those of strong conflicts (*UF*_*AV*_ >1) and large-size multi-project instances are shown with * sign and underline, respectively. As can be seen in Table 4, for 15 out of 20 subsets, the proposed approach performs best. Specifically, for 12 out of 14 subsets with strong conflicts and for 7 out of 8 subsets with large-size multi-project instances, the proposed approach obtains a much lower *TTC* than any other methods. To clearly show the better performance with regard to the instance size and resource utilization factor, we compute the percentage improvement of our method comparing with CMAS/ES and CMAS/SA, respectively. The statistical comparisons for small- and large-size instances, and for *UF*_*AV*_ <1 and *UF*_*AV*_ >1 instances are depicted in Fig 11 and Fig 12, respectively. In each figure, the maximum and the average of percentage improvements are demonstrated on the left (a) and right (b), respectively. It is indicated that for both CMAS/ES and CMAS/SA, our method shows superior performance on large-size and strong conflicts instances. Moreover, for all subsets, the percentage improvements of our approach in reducing *TTC* achieve an average of 10%-15% compared to CMAS/ES, and a maximal of 20%-36% compared to CMAS/SA. From above results, the two-stage decomposition approach outperforms other decentralized methods in providing satisfactory solutions in terms of *TTC*, especially for large-size and strong conflicts instances.

**Table 4:** Comparisons of *TTC* among different decentralized methods.

Problem subset	Problem size	*UF*_*AV*_	Two-stage decomposition	CMAS/ES	CMAS/SA
MP30_2	60	0.84	**412.6**	529.4	527.8
MP90_2	180	0.57	890.4	**752.8**	790
MP120_2	240	1.31	11767.8*	12470.4	12017.6
MP30_5	150	0.82	**1486.4**	1612.4	1737
MP90_5	450	0.61	2827.4	3959.6	**2687.6**
MP120_5	600	1.32	21205	23118	20232.6*
MP30_10	300	2.38	9461*	11920.4	12221.4
MP90_10	900	1.14	18879*	20509.8	19799.2
MP120_10	1200	1.91	76361.2*	82799.8	119064
MP30_20	600	3.37	58972.6*	69797.4	68681.6
MP90_20	1800	0.90	32046.8	33716.2	**29935.4**
MP120_20	2400	0.87	**41850.4**	52210	NA
MP90_2AC	180	2.27	10394*	10585.6	10944.75
MP120_2AC	240	1.36	6796.7	6925.4	5735.2*
MP90_5AC	450	4.99	56614.9*	62085.3	59998.9
MP120_5AC	600	3.80	110993.3*	135607.3	130062.9
MP90_10AC	900	3.85	101098.8*	122450.8	117388.2
MP120_10AC	1200	2.61	117991.9*	153229.9	147253.8
MP90_20AC	1800	2.70	77437.4*	94605.6	88618
MP120_20AC	2400	3.65	419621.6*	520681.9	500280.1

NA indicates some schedule results of multi-project instances in MP120_20 subset are not available on MPSPLIB web site.

## Conclusion and future work

This paper is devoted to solving DRCMPSP using a two-stage decomposition approach. Individual project managers focus on minimizing the project completion time initially while the senior manager pays more attention to reducing *TTC* from a global perspective. A FBHGA is introduced to determine the local initial schedule for each *PA* in stage one, and a sequential game-based negotiation mechanism is proposed to coordinate the global resources allocation among multiple projects in stage two.

The performance of the proposed approach has been evaluated on the MPSPLIB benchmark. High-quality local initial schedules can be generated by FBHGA for every individual *PA* and the average *ARD* with respect to the optimal solutions over all problem subsets is less than 1%. By conducting preliminary experiments on all subsets, satisfactory solutions in terms of *TTC* can be generated in reasonable CPU running time after 10 iterations of sequential game-based negotiation. The senior manager will face complex multi-project management situation and more *TTC* will be incurred when the shared global resources are relatively scarce. The performance of our approach is compared with two existing decentralized methods with different coordination mechanisms. Experiments reveal that the proposed approach with sequential game-based negotiation mechanism can significantly reduce *TTC*, especially for the instances with large problem size and high resource utilization factor.

As the increasing of multinational corporations in the process of globalization, more and more multiple projects are executed simultaneously at different locations. Our proposed two-stage decomposition approach provides decision guidance for distributed multi-project scheduling managers. Meanwhile, the sequential game-based negotiation mechanism opens a new perspective for shared resource allocation. However, there is a limitation that the randomized search heuristic procedure is used to obtain a best subgame perfect Nash equilibrium result. In the future, we aim to find the approximate optimal solutions by efficient meta-heuristics. In addition, considering the possible uncertain environments in today’s globally active industries, the proposed two-stage decomposition approach with sequential game-based negotiation mechanism opens up new directions for stochastic distributed multi-project scheduling problem.

## Supporting information

S1 Appendix Multi-project instances. (ZIP)
